# Impact of Foliar Fertilization on the Content of Silicon and Macronutrients in Sugar Beet

**DOI:** 10.3390/plants8050136

**Published:** 2019-05-22

**Authors:** Arkadiusz Artyszak, Dariusz Gozdowski, Katarzyna Kucińska

**Affiliations:** 1 Department of Agronomy, Warsaw University of Life Sciences—SGGW, 159 Nowoursynowska St., 02-776 Warsaw, Poland; katarzyna_kucinska@sggw.pl; 2 Department of Experimental Design and Bioinformatics, Warsaw University of Life Sciences—SGGW, 159 Nowoursynowska St., 02-776 Warsaw, Poland; dariusz_gozdowski@sggw.pl

**Keywords:** *Beta vulgaris*, macronutrients, pure sugar yield, silicon uptake

## Abstract

The present study was conducted to assess the effect of various multinutrient foliar fertilization treatments on nutrient accumulation in the roots and leaves of sugar beet. The field experiment was performed in two growing seasons (2013 and 2014) in the southeastern region of Poland. The experiment was conducted as a randomized block design with four replications. To determine the content of the selected nutrients (Si, N, P, K, Mg, and Ca), samples of leaves and roots were collected during harvest. Fertilization treatments applied on the plants’ leaves affected the silicon (Si) content in the roots of sugar beet and the total Si uptake. Moreover, foliar fertilization had a significant effect on the P content in the leaves and the N and K contents in the roots. The Si content in the sugar beet leaves and roots ranged from 0.55 to 1.97 g kg^−1^ dry matter (d. m.) and from 0.49 to 1.59 g kg^−1^ d. m., respectively. The total Si uptake ranged from 20.3 to 46.7 kg ha^−1^. Most of the Si content taken up with each fertilization variant was accumulated in the roots. The yield of pure sugar was not correlated with the Si content in the leaves and roots.

## 1. Introduction

Although silicon (Si) is the most abundant mineral element in the soil, it is not considered as an essential element for plant growth. Beneficial effects of Si on rice growth are attributable to the characteristics of a silica gel that is accumulated on the epidermal tissues in rice. These effects are observed most clearly under high-density cultivation systems with heavy applications of nitrogen fertilizers. Si is therefore now recognized as an “agronomically essential element” in Japan [1]. Recently, Si has gained global attention because it induces disease and pest resistance in plants and can reduce doses of pesticides applied for plant protection. Si is also considered as an environmentally safe element [1]. The application of Si has been found to be beneficial for the growth, development, and yield of various plants and to alleviate various stresses such as nutrient imbalance. Moreover, the addition of Si improves organogenesis, embryogenesis, growth traits, and the morphological, anatomical, and physiological characteristics of leaves; enhances tolerance to low temperature and salinity; protects cells against metal toxicity; prevents oxidative phenolic browning; and reduces the incidence of hyperhydricity in various plants species [2]. Si fertilization by using natural silicates has shown the potential to mitigate environmental stresses and soil nutrient depletion and is therefore an alternative to the extensive use of phytosanitary measures and NPK fertilizers for maintaining sustainable agriculture [3,4]. However, the Si concentration in plants depends more on the phylogenetic position of the plant than on its environment (i.e., the Si concentration in the soil and the pH of the soil solution). Unlike other elements, Si is abundant in nearly all soils; therefore, environmental criteria do not affect Si accumulation in plants. Moreover, many plants are not able to accumulate Si at sufficiently high levels for it to be beneficial [5]. Plants can uptake Si from soil, but the content of Si available to them depends on many factors, such as pH of the soil, availability of micronutrients in soil, and aluminum and heavy metal content [6]. A better understanding of the Si uptake capacity might help to enable such plants to accumulate more Si and to improve their ability to overcome biotic and abiotic stresses. Despite the important role of Si in plants, there is still a lack of data on the effect of sugar beet fertilization on Si uptake from soil. There is only one report on Si content in sugar beet [7]. The beneficial effect of Si on the yield of some crops, such as sugar beet [8,9,10,11,12,13], lupine and pea [14], potato [15,16], and seeds of grasses [17], was recently reported. A comprehensive review on the beneficial effect of Si on crop yield in Europe has been published by Artyszak [18]. In the available literature, there are no results that can answer the question of whether the foliar application of macro- and micronutrients can significantly affect Si content in sugar beet plants. Therefore, it was hypothesized that foliar nutrition of macro- and microelements has a significant impact on the chemical content of plants as well as on the content of Si in leaves and roots and its uptake by sugar beet plants. The aim of the study was to determine the effect of different foliar applications of multicomponent preparations on the accumulation of Si and some macronutrients in the leaves and roots of sugar beet.

## 2. Results

The Si content in the sugar beet leaves and roots ranged from 0.55 to 1.97 g kg^−1^ dry mass (d. m.) and from 0.49 to 1.59 g kg^−1^ d. m., respectively (Table 1).

Foliar fertilization did not significantly affect the Si content in the sugar beet leaves. However, foliar fertilization in three of the fertilization variants resulted in a significantly lower Si content in sugar beet taproots than in the control plants. The applied foliar fertilization with macro- and micronutrients affected the P content in the leaves and dry matter (d. m.) and the N and K content in the taproots (Table 1). Sugar beet plants fertilized by fertilization treatment no. 4 had the highest P content in the leaves and the highest K content in the roots. The control variant had the highest content of d. m. and the lowest N content in the roots. 

The total Si uptake ranged from 20.3 to 46.7 kg ha^−1^ according to the fertilization treatment (Table 2). Most of the Si taken up with each fertilization variant was accumulated in the roots (Table 3). The results of the effect of foliar fertilization on the macronutrient contents in the sugar beet roots and leaves were ambiguous.

The variability of the Si content in the leaves was 69.0%, which was greater than that in the roots (48.1%). The variability of the Si uptake and deposition was similar between the leaves (70.1%) and the roots (51.3%) (Table 4). 

With regard to d. m. and macronutrient contents, the smallest variability was observed for the P content in the leaves and for the d. m. in the roots. However, the smallest coefficient of variation values for the macronutrient uptake were observed for P in the leaves and for K in the roots. 

The results showed that the pure sugar yield (biological yield of sugar reduced by loss of sugar in molasses) was not correlated with the Si content in both the leaves and roots (Table 5). 

However, some important correlations were observed in these experiments. The technological sugar yield showed a negative correlation with the K content in the leaves, but showed positive correlations with the Mg content in the leaves and the P content in the roots. These correlations showed that foliar fertilization with Mg had positive effects on the technological sugar yield. 

After removing variables without significant effects, the regression equations defining the relationship of the technological sugar yield with the contents of the analyzed nutrients in the leaves and roots were as follows: 

Pure sugar yield = 20.611 − 0.142 × content of K in leaves, R^2^ = 0.67; 

Pure sugar yield = 12.476 + 0.330 × content of Mg in leaves, R^2^ = 0.25; 

Pure sugar yield = 13.244 + 4.454 × content of P in roots, R^2^ = 0.29. 

## 3. Discussion

As compared to other crops, the Si content in sugar beet is relatively low. Guntzer et al. estimated the Si content in sugar beet leaves to be 2.34–7% d. m. [3]. For this calculation, they used data from a study by Harland et al. [19], who reported that the silicate content of fresh fodder sugar leaves was 5–15% of d. m. Such a high silicate content in fodder sugar beet leaves may be attributed to the fact that the leaves were acquired by mechanical harvesting or that they contained some amount of silicates from the soil. Guntzer et al. [3] calculated the Si content in the silicate (SiO_2_) content based on the atomic weights of Si and O. Our results did not confirm such high Si contents in any part of the sugar beet plants, because the average contents of this element in the leaves and roots were 1.26 and 0.93 g kg^−1^ d. m., respectively, which is five times less than that reported by the above-cited authors and similar to or slightly higher than the values obtained in other experiment.It is worth emphasizing that both our experiments presented and published in 2018 were carried out in the same location and in the same years (2013–2014), but in different soil conditions in the year 2013. Si content in the leaves of sugar beet plants fertilized with foliar marine calcite and foliar silicon were, respectively, 0.59–1.04 and 0.67–0.80 g Si kg^−1^ (control = 0.84 g Si kg^−1^). Si content in the roots of sugar beet fertilized with foliar marine calcite and foliar silicon were, respectively, 1.13–2.08 and 0.82–1.18 g Si kg^−1^ (control = 1.05 g Si kg^−1^) [7].

In the presented research, the Si uptake in the leaves was much greater than that obtained in the experiment where fertilizers with silicon were applied [7] In that study, the intake of Si stored in the leaves of sugar beet plants fertilized with foliar marine calcite or foliar silicon was, respectively, 4.10–4.33 and 3.62–5.57 kg ha^−1^ (control = 4.86 kg ha^−1^). However, in the presented study, the intake of Si stored in the roots was usually smaller than that observed in the experiment with silicon fertilizers where the authors found that the uptake of Si in the roots of sugar beet fertilized by foliar marine calcite or foliar silicon was, respectively, 26.5–58.2 and 20.3–3.3 kg ha^−1^ (control = 24.8 kg ha^−1^). For this reason, total Si uptake in the presented research depended on the fertilization type, and its value is close to or less than that reported by those authors. They observed that the total silicon uptake (leaf and root) by sugar beet plants fertilized with foliar marine calcite or foliar silicone was, respectively, 30.6–62.5 and 23.9–36.9 kg ha^−1^ (control = 29.6 kg ha^−1^) [7].

In the present study, the variability of Si content in sugar beet leaves was higher (coefficient of variation (CV) = 69.0%) than that in the roots (CV = 48.1%). Opposite results were observed in the experiment with fertilizers with silicon where was found that the variability of Si content in the sugar beet leaves was lower (CV = 55.1%) than that in the roots (CV = 63.2%). Similarly, in the present study, the variability of Si uptake stored in the leaves (CV = 71.0%) was higher than that stored in the roots (CV = 51.0%). However, Artyszak et al. found that the variation in silicon uptake stored in sugar beet leaves was lower (CV = 48.6%) than that stored in the roots (CV = 74.9%) [7].

The technological sugar yield was negatively correlated with the K content in the leaves and positively correlated with the Mg content in the leaves and the P content in the roots. The authors found that the technological sugar yield was positively correlated with the Si and K contents in the leaves and with the Mg content in the roots. Although in the present study, it was not possible to determine a significant relationship between the technological sugar yield and the Si content in the leaves, the correlation coefficient assumed quite a high value of 0.50 [7].

The use of foliar Si-free fertilizers did not significantly affect the content of macroelements in sugar beet plants and their uptake in most types of fertilization. However, in the study of these authors, foliar fertilization containing silicon significantly affected the content of d. m., Mg, and Ca in the leaves and the content of d. m. and N in the roots [7]. 

As noted in previous studies, the smallest variability in d. m. content was observed in both leaves and roots. The lowest CV value in nutrient uptake was observed for N stored in leaves and roots. In the present study, the variability of Si content in leaves was higher (CV = 68.96%) than that in roots (CV = 48.08%). Similarly, the value of CV for Si accumulated in leaves and roots was 70.95% and 51.3%, respectively. These results are different from those previously reported, where the variability of Si content in leaves and roots was significantly lower at 26.3% and 37.9%, respectively. The value of CV for Si accumulated in leaves and roots was 43.2% and 27.0%, respectively [7].

The correlations of technological sugar yield with macroelement contents observed in the present study were different from those, where the authors found a positive correlation of the sugar yield with the content of d. m. and P in leaves and the content of P, K, and Mg in the roots [7].

Many studies indicate the importance of Si as an element that facilitates the uptake of macro- and micronutrients and protects plants against excessive accumulation of heavy metals and nonmetallic ions [20]. However, there is still a lack of research explaining what factors facilitate or reduce Si uptake. Foliar nutrition with Si-free foliar fertilizers did not positively affect either the Si content in sugar beet plants or the uptake of this nutrient from the soil. However, the significantly higher Si content in the roots and the highest uptake of this component by the control plants (without fertilization) suggest that the easy availability of other nutrients from foliar fertilizers does not facilitate uptake and accumulation of Si. Thus, the hypothesis that foliar fertilization can favorably affect Si uptake from the soil has not been confirmed. 

Requirement of mineral elements, including Si, differs with different plant organs and tissues. Si content in crop plants is very diverse and it does not depend only on the species. Plants of the same species also differ in the content of this ingredient [20]. This has been confirmed by our research on sugar beet. A better understanding of the mechanisms and conditions of Si uptake could facilitate the beneficial effect of Si on plants. Some plant species (e.g., rice) can even hyperaccumulate Si. Accumulation of more than 10% Si of the dry mass in the rice husk is required for protecting the grains from water loss and pathogen infection [21]. Si content in potato (*Solanum tuberosum* L.) tubers ranged from 209 to 479 mg kg^−1^, depending on the potato variety [22]. Different species or cultivars grown at various concentrations of Si absorb different amounts of Si, as reported for vegetables, fruits, and rice [20,23]. Under drought-stressed conditions, the Si content in potato leaves was 0.41% d. m. (without Si fertilization) and 0.47% d. m. after Si fertilization [24]. However, under normal conditions (without drought), the Si contents were lower: 0.37% and 0.42%, respectively. The effects of various fertilization methods on the contents of Si and other nutrients in cucumber plants was observed [25], as well as the Si content in the soybean leaves (*Glycine max* (L.) Merr.) was higher after the application of silicate to the soil [26]. The Si content increased from 2.64 to 3.70 g Si kg^−1^ [26]. Similarly, in corn leaves (*Zea mays* L.), the Si content increased from 10.0 to 11.8 g Si kg^−1^ after fertilization with silicate [26]. Many studies have shown the beneficial effect of Si fertilization on its higher content in different plant tissues [20]. In other studies, N fertilization of maize and mallow had a significant effect on Si content in silage made from these crops. These authors showed a significantly lower content of Si in silage made of plants fertilized with high doses of N [27]. This confirms our own observations that the easy availability of other elements for plants does not favor Si accumulation. 

There are other observed benefits of increased Si accumulation in plants; for example, increase of K concentration in leaves, stems, and roots and alleviation of K deficiency in soybean seedlings after Si addition to the K-deficient seedling growth medium [28]. Some phytoextracts applied with Si fertilizer improved the Si contents in roots and leaves of salt-stressed pea plants [29]. In another study with alfalfa, Si fertilization significantly increased the leaf area, plant height, forage yield, and shoots per plant during the reproductive period. Si also increased the root volume of secondary roots and the root biomass [30]. One of the most recent studies on Si fertilization in potted alfalfa (*Medicago sativa*) reported that the effect of Si fertilization is ambiguous and can be modified by other factors. The addition of Si increased the P and K contents of alfalfa shoots when accompanied by an increase in the P content and a decrease in the K content in the soil. Si increased the total content of K and P but had no effect on the total N content in the alfalfa shoots [31].

The crop with the highest Si uptake (approximately 500 kg Si ha^−1^ year^−1^) is rice [32]. Si uptake in 46 crops grown hydroponically showed a general pattern of Si deposition along the leaf margins and in the leaf trichomes. Minimal Si was found in the roots and stems [33]. Similarly, the salinity-induced reduction in K content was partially ameliorated by Si application, particularly in the roots of the common bean (*Phaseolus vulgaris* L.) [34]. Our research has not shown an effect of Si content in the leaves and the roots on the pure sugar yield. In a previous study on potatoes, Si content was not correlated with some yield features [35]. Eneji et al. also observed correlations between Si and P uptake [36]. Similar results for microelements were reported by Hernandez-Apaola [37]. According to Mali and Aery, the P uptake in both hydroponics and the soil was improved even at low Si concentrations through the activation of H-ATPase [38]. These authors also observed better absorption of N and Ca for cowpea and wheat fertilized with increasing doses of sodium metasilicate (50–800 mg Si kg^−1^) as well as better nodulation and markedly better N_2_ fixation in cowpea [38,39].

Our results on sugar beet, as well as those obtained by other authors for different species accumulating large and small amounts of Si, suggest the need for further research on the effects of agronomic factors on the uptake and storage of Si. Given that Si is the only element that confers resistance to multiple stresses, affects yields and quality, and is nontoxic to humans and environment, the use of this element as a biostimulant together with fertilizers in field crops and in horticulture is expected to increase considerably in the future [20].

## 4. Materials and Methods 

The study was conducted in Sahryń (50°41’ N and 23°46’ E), southeastern Poland, in two growing seasons (2013 and 2014). In each season, a randomized complete block design experiment with four replications was conducted. Following the recommendations of the fertilizer producers, seven treatments were tested, including six types of foliar fertilizations and a control treatment. Foliar fertilization types were tested in accordance with the recommendations of the producers of the fertilizers. Such types of fertilization are mostly used in agronomic practice in Poland. The fertilizers were applied once at the 4–6 leaf stage of sugar beet (treatments 1 and 4); twice at the 4–6 leaf stage and one week later (treatments 2 and 5); and thrice at the 4–6 leaf stage, one week later, and then two weeks later (treatments 3 and 6). The numbers of fertilization types, the time of their application, and the name of the preparations are described in Table 6, while the total amounts of nutrients applied are presented in Table 7.

The compositions of fertilizers (g dm^−3^) were as follows: (1) FoliQ Ascovigor: N—317, K—158, Ca—129, S—102, B—38.1, Cu—0.004, I—0.04, Fe—0.06, Mn—10.2, Zn—6.4, *Ascophyllum* marine algae extract, natural growth hormones (cytokinins, auxins, gibberellins, betanin), vitamins; (2) FoliQ Mg: N—56, Mg—127, S—56; (3) FoliQ Mikromix: N—78, K—130, Mg—28.3, S—34.8, B—4.7, Cu—7.9, Fe—15.7, Mn—23.6, Mo—0.15, Zn—15.7; (4) FoliQ B: B—150; (5) FoliQ Zn+: N—54.8, B—54.8, Zn—54.8; (6) FoliQ Kombimax: N—300, K—187, Mg—36.2, S—10.0, B—0.30, Cu—0.75, Fe—1.50, Mn—0.75, Mo—0.15, Zn—0.75.

Precipitation during the growing period, from the beginning of April until the end of October, was 387 mm and 550 mm in seasons 2013 and 2014, respectively. The average temperature of the two growth seasons was very similar (14.8 °C and 14.5 °C, respectively). The soil type was Calcic Chernozem (Aric, Loamic) containing 1.1% organic carbon in both years (pH range: 7.2–7.4) [40]. The content of the available forms of P was 109 and 22 mg kg^−1^, and the content of K was 95 and 75 mg kg^−1^ in the subsequent seasons (2013 and 2014, respectively) [41,42]. The sugar beet (*Beta vulgaris* L. subsp. *vulgaris* var. *altissima* Doll) variety Primadonna KWS was cultivated after winter rape cultivation. Fertilizers (NPK) in the solid form were applied before plowing in the autumn. The doses were adjusted to the soil fertility and the expected yields. Total rates of macronutrients were as follows (ha^−1^): 135 kg N, 39 kg P, 112 kg K, 13 kg S, 9.6 kg MgO, and 5.7 kg Ca. More details about the weather and soil conditions and the agricultural practices were reported by Artyszak et al. [43]. For the chemical analyses and to determine the d. m. content, representative samples of roots and leaves from the center of the rosettes were harvested from each plot during harvesting. The d. m. content was determined by the oven-drying method. The contents of nutrients such as N, P, K, Ca, and Mg were determined in the oven-dried plant material after the wet digestion of the samples with sulfuric acid and the addition of 30% hydrogen peroxide solution. The nutrient content was determined by the following methods: N by direct potentiometric titration with sodium hypobromite (Kjeldahl method); P by the spectrophotometric method with a solution of nitric acid, ammonium metavanadate, and ammonium molybdate at the wavelength of 470 nm; K, Ca, and Mg were determined by atomic absorption spectrometry at 766.5, 422.7, and 285.2 nm, respectively [44,45,46,47,48]. The Si content was determined by two-phase wet digestion according to the method of Kraska and Breitenbeck [49].

Sucrose, alpha-amino nitrogen, Na, and K contents were determined using the automatic beet analysis system “Venema” by Kutno Sugar Beet Breeding Station Ltd. in Straszków, Poland. A more detailed description is reported in [43]. 

Pure sugar yield was calculated from the following formula [50]: 

Pure sugar yield (t ha^−1^) = Root yield (t ha^−1^) × [sugar content (%) − loss of sugar efficiency (%)]

Sugar efficiency loss (%) = Standard loss of molasses (%) + 0.6 (%); 

Standard loss of molasses (%) = 0.012 × (K + Na) + 0.024 (α-amino-N) + 0.48; 

where K, Na, and α-amino-N content are in mmol kg^−1^ of the pulp.

The yields of leaf d. m. and root d. m. were calculated as the products of the yield and the d. m. content. The uptake of N, P, K, Mg, Ca, and Si was calculated from their content in plants and the d. m. yield. The experimental data were statistically analyzed by one-way analysis of variance, and the mean values were compared using the least significant difference, with the level of significance of α = 0.05. Statistical analyses were performed with SAS 9.1 software (Cary, USA) by using the GLM (General Linear Model) procedure. The evaluation of the correlation between the measured traits was calculated using simple Pearson’s correlation coefficients. The significance of correlations was assessed at *p* ≤ 0.05 and *p* ≤ 0.01. Relationships between the pure sugar yield and the macronutrients and Si content in leaves and roots were evaluated using multiple regression with backward selection of variables.

## 5. Conclusions

The Si content in the leaves and roots of sugar beet for all the treatments ranged from 0.55 to 1.97 and from 0.49 to 1.59 g kg^−1^ d. m., respectively. The foliar application of macro- and micronutrients had no significant effect on the Si content in the leaves. Sugar beet plants accumulated 4.7–15.6 and 12.5–35.8 kg Si ha^−1^ in the leaves and roots, respectively, and a total of 20.3–46.7 kg Si ha^−1^ in the entire plant. Most of the Si taken up with each fertilization variant was accumulated in the roots.

Correlations between the Si content in the leaves and that in the roots with the pure sugar yield were not significant. The Mg content in leaves and the P content in the roots were positively correlated with the pure sugar yield. However, a negative correlation with the pure sugar yield was found for the K content in the leaves.

## Figures and Tables

**Table 1:** The effect of foliar fertilization with macro- and micronutrients on the chemical content of sugar beet leaves and roots (mean values from 2013 to 2014).

Fertilization Treatment	Dry Matter (%)	g kg^−1^ Dry Matter (d. m.)					
		Si	N	P	K	Mg	Ca
Leaves							
0	14.67 a ^1^	1.66 a	29.45 a	1.80 ab	40.45 a	10.50 a	23.55 a
1	14.03 a	0.90 a	29.30 a	1.80 ab	36.20 a	12.00 a	22.90 a
2	14.05 a	0.98 a	30.33 a	1.87 ab	35.00 a	11.00 a	22.20 a
3	14.34 a	0.96 a	30.38 a	1.90 ab	33.40 a	16.00 a	28.40 a
4	14.39 a	0.55 a	32.60 a	1.95 b	29.50 a	13.50 a	26.45 a
5	14.08 a	1.82 a	33.60 a	1.75 a	28.60 a	16.50 a	25.55 a
6	14.31 a	1.97 a	29.75 a	1.77 ab	28.85 a	14.50 a	24.50 a
Roots							
0	25.01 c	1.59 b	2.85 a	0.90 a	4.80 ab	1.40 a	3.40 a
1	24.45 abc	0.52 a	4.40 ab	0.55 a	4.65 ab	1.10 a	2.00 a
2	22.92 a	0.49 a	4.90 ab	0.85 a	3.30 a	1.40 a	2.70 a
3	24.50 bc	0.95 ab	4.20 ab	0.90 a	4.35 ab	1.35 a	2.10 a
4	24.14 abc	1.02 ab	5.00 ab	0.95 a	5.40 b	1.45 a	1.90 a
5	24.01 abc	0.74 a	4.85 ab	0.70 a	4.40 ab	1.30 a	3.55 a
6	23.83 ab	1.18 ab	6.35 b	0.90 a	4.30 ab	1.40 a	2.20 a

^1^ Different letters in columns indicate significant differences between mean values for fertilization variants at the 0.05 probability level.

**Table 2:** Nutrient uptake by sugar beet plants (mean values from 2013 to 2014).

Fertilization Treatment	Si	N	P	K	Mg	Ca
	kg ha^−1^					
Leaves						
0	10.9 ab ^1^	207.9 a	12.5 ab	287.7 a	71.7 a	160.3 a
1	6.9 ab	216.0 a	13.2 ab	256.1 a	91.2 a	171.1 a
2	7.9 ab	231.0 a	14.2 ab	255.2 a	85.9 a	169.8 a
3	7.7 ab	237.7 a	14.8 ab	245.5 a	130.7 a	225.5 a
4	4.7 a	279.2 a	16.5 b	238.0 a	115.4 a	216.7 a
5	12.0 ab	211.1 a	11.0 a	175.5 a	104.2 a	160.8 a
6	15.6 b	226.2 a	13.3 ab	214.1 a	111.6 a	186.6 a
Roots						
0	35.8 b	63.3 a	21.5 a	108.2 ab	31.8 a	79.7 a
1	13.4 a	110.6 ab	14.1 a	114.4 ab	26.8 a	48.3 a
2	12.5 a	123.3 ab	21.7 a	82.5 a	35.4 a	68.0 a
3	25.6 ab	111.4 ab	24.2 a	115.7 ab	35.5 a	55.8 a
4	26.3 ab	127.3 ab	23.8 a	135.0 b	36.5 a	48.5 a
5	17.7 ab	112.6 ab	17.3 a	113.4 ab	33.8 a	85.1 a
6	31.0 ab	155.3 b	23.0 a	107.6 ab	35.1 a	53.8 a
Leaves + roots						
0	46.7 b	271.2 a	34.0 abc	395.9 d	103.5 a	240.0 ab
1	20.3 a	326.6 a	27.3 a	370.5 cd	118.0 ab	219.4 a
2	20.4 a	354.3 a	35.9 abc	337.7 bc	121.3 ab	237.8 ab
3	33.3 ab	349.1 a	39.0 bc	361.2 cd	166.2 b	281.3 c
4	30.9 ab	406.5 a	40.3 c	373.0 d	151.9 ab	265.2 bc
5	29.7 ab	323.7 a	28.3 ab	288.9 a	138.0 ab	245.9 ab
6	46.5 ab	381.5 a	36.3 abc	321.7 ab	146.7 ab	240.4 ab

^1^ Different letters in columns indicate significant differences between mean values for variants at the 0.05 probability level.

**Table 3:** The distribution of Si and macronutrients in sugar beet plants (mean values from 2013 to 2014).

Fertilization Treatment	Si	N	P	K	Mg	Ca
	%					
Leaves						
0	23.3 ab ^1^	75.8 d	41.7 a	70.7 c	69.3 a	67.9 b
1	34.0 bc	66.3 bc	50.1 b	67.0 b	73.2 b	76.4 d
2	38.7 c	65.7 bc	41.9 a	74.0 d	70.2 a	71.7 c
3	23.1 ab	68.0 bc	38.9 a	66.0 b	75.3 bc	79.7 ef
4	15.2 a	68.4 c	40.6 a	63.8 ab	75.5 bc	81.8 f
5	40.4 c	65.1 b	39.0 a	62.2 a	76.8 c	65.0 a
6	33.5 bc	59.2 a	38.7 a	65.3 ab	75.6 bc	77.4 de
Roots						
0	76.7 bc	24.2 a	58.3 b	29.3 b	30.7 c	32.1 e
1	66.0 ab	33.7 bc	49.9 a	33.0 c	26.8 b	23.6 c
2	61.3 a	34.3 bc	58.1 b	26.0 a	29.8 c	28.3 d
3	76.9 bc	32.0 bc	61.1 b	34.0 c	24.7 ab	20.3 ab
4	84.8 c	31.6 b	59.4 b	36.2 cd	24.5 ab	18.2 a
5	59.6 a	34.9 c	61.0 b	37.8 d	23.2 a	35.0 f
6	66.5 ab	40.8 d	61.3 b	34.7 cd	24.4 ab	22.6 bc

^1^ Different letters in columns indicate significant differences between mean values for variants at the 0.05 probability level.

**Table 4:** Variability parameters of the nutrient content and uptake in sugar beet leaves and roots in 2013 and 2014.

	Mean	Range	Coefficient of Variation (%)	Mean	Range	Coefficient of Variation (%)
	Leaves			Roots		
Dry matter (%)	14.27	12.62–16.23	6.18	24.12	15.8–26.3	6.66
	Content of elements in dry matter (g kg^−1^)					
Si	1.26	0.48–3.27	68.96	0.93	0.32–1.71	48.08
N	30.77	24.5–34.4	8.91	4.65	2.3–6.9	28.06
P	1.83	1.63–2.00	5.09	0.82	0.4–1.3	32.1
K	33.14	18.9–51.6	37.9	4.46	3.1–5.8	18.5
Mg	13.43	7.0–22.0	31.82	1.34	0.9–1.7	18.17
Ca	24.79	18.0–32.6	16.93	2.55	1.5–4.3	32.73
	Uptake (kg ha^−1^)					
Si	9.37	3.28–26.35	70.95	23.2	7.0–47.6	51.3
N	229.8	152.2–347.4	21.5	114.8	58.5–164.9	29.5
P	13.6	9.7–19.4	17.7	20.8	8.8–33.1	39.9
K	238.9	153.9–385.2	33.4	111	67.8–159	24.7
Mg	101.5	47.0–193.6	41.3	33.5	18.5–49.1	26.4
Ca	184.4	120.9–286.9	24.1	62.7	38.1–109.4	32.8
Allocation in plants (%)						
Si	29.7	9.3–48.7	24.7	70.3	51.3–90.7	26.4
N	66.9	55.5–80.8	9.6	33.1	19.2–44.5	19.4
P	41.6	21.6–61.1	22.9	58.4	38.9–78.4	16.3
K	67.0	49.5–84.7	15.6	33.0	15.3–50.5	31.6
Mg	73.7	59.0–85.7	9.4	26.3	14.3–41.0	26.3
Ca	74.3	56.2–87.2	10.3	25.7	12.8–43.8	29.8

**Table 5:** Correlation coefficients of pure sugar yield and chemical content of sugar beet plants in 2013 and 2014 (*n* = 14).

	Pure Sugar Yield (t ha^−1^) vs. Dry Matter and Content of Elements in Leaves	Pure Sugar Yield (t ha^−1^) vs. Dry Matter and Content of Elements in Roots
d. m.	−0.38	0.09
Si	0.50	0.09
N	0.16	0.02
P	−0.23	0.54 *
K	−0.82 **	0.07
Mg	0.65 *	0.38
Ca	0.32	−0.19

* Significant correlation at *p* ≤ 0.05; ** significant correlation at *p* ≤ 0.01.

**Table 6:** Types of foliar fertilization for sugar beet tested in the experiment.

Number of Fertilization Type	Names of Preparations		
	Treatment at the 4–6 Leaf Stage of Sugar Beet	Treatment One Week Later	Treatment Two Weeks after First Treatment
1	FoliQ Ascovigor + FoliQ Mg	-	-
2	FoliQ Ascovigor + FoliQ Mg	FoliQ Mikromix + FoliQ B	-
3	FoliQ Ascovigor + FoliQ Mg	FoliQ Mikromix + FoliQ B	FoliQ Zn+
4	FoliQ Ascovigor + FoliQ B	-	-
5	FoliQ Ascovigor + FoliQ B	FoliQ B + FoliQ Kombimax	-
6	FoliQ Ascovigor + FoliQ B	FoliQ B + FoliQ Kombimax	FoliQ Zn+

**Table 7:** The total doses (g ha^−1^) of nutrients applied by foliar fertilization in the investigated treatments of sugar beet (data provided by the fertilizer producers).

Application of Fertilizers	Control (0)	Type of Fertilization					
		1	2	3	4	5	6
N	-	802	880	990	951	1851	1961
K	-	316	446	446	474	1035	1035
Mg	-	381	409	409	-	109	109
Ca	-	258	258	258	387	387	387
S	-	372	407	407	306	336	336
B	-	76	231	341	339	565	675
Cu	-	0.01	7.91	7.91	0.01	2.26	2.26
I	-	0.08	0.08	0.08	0.12	0.12	0.12
Fe	-	0.12	15.82	15.82	0.18	4.68	4.68
Mn	-	20.4	44.0	44.0	30.6	32.9	32.9
Mo	-	-	0.15	0.15	-	0.45	0.45
Zn	-	12.8	28.5	138.1	19.2	21.5	131.1

## Abbreviations

d. m.—dry matter, CV—coefficient of variation.

## References

[1] Ma J.F., Takahasi E. (2002). Soil, Fertilizer, and Plant Silicon Research in Japan.

[2] Sivanesan I., Park S.W. (2014). The role of silicon in plant tissue culture. Front. Plant Sci..

[3] Guntzer F., Keller C., Meunier J.D. (2012). Benefits of plant silicon for crops: A review. Agron. Sustain. Dev..

[4] Elsokkary I.H. (2018). Silicon as a beneficial element and as an essential plant nutrient: An outlook. Alex. Sci. Exch. J..

[5] Ma J.F., Yamaji N.A. (2015). Cooperative system of silicon transport in plants. Trends Plant Sci..

[6] Szulc W., Rutkowska B., Hoch M., Spychaj-Fabisiak E., Murawska B. (2015). Exchangeable silicon content of soil in a long-term fertilization experiment. Plant Soil Environ..

[7] Artyszak A., Gozdowski D., Kucińska K. (2018). Effect of foliar fertilization using silicon and calcium or only silicon on chemical composition of sugar beet. Listy Cukrov. A Řepařské.

[8] Artyszak A. (2017). Possibilities of Using Silicon for Foliar Fertilization of Sugar Beet.

[9] Artyszak A., Gozdowski D., Kucińska K. (2014). The effect of foliar fertilization with marine calcite in sugar beet. Plant Soil Environ..

[10] Artyszak A., Gozdowski D., Kucińska K. (2015). The effect of silicon foliar fertilization in sugar beet—*Beta vulgaris* (L.) ssp. *vulgaris* conv. *crassa* (Alef.) prov. *altissima* (Döll). Turk. J. Field Crop..

[11] Artyszak A., Gozdowski D., Kucińska K. (2016). The effect of calcium and silicon foliar fertilization in sugar beet. Sugar Tech..

[12] Hřivna L., Hernandez Kong J., Machálková L., Burešová I., Sapáková E., Kučerová J., Šottníková V. (2017). Effect of foliar nutrition of potassium and silicon on yield and quality of sugar beet in unusual windy conditions in 2014 and 2015. Listy Cukrov. A Řepařské.

[13] Urban J., Pulkrábek J. (2018). Increased yield and quality of sugar beet by means of foliar nutrition and biologically active substances. Listy Cukrov. A Řepařské.

[14] Sulewska H., Ratajczak K., Niewiadomska A., Koziara W., Panasiewicz K., Faligowska A. (2018). Titanium, silicon, boron, zinc and molybdenum-containing formulations in the white lupine and pea cultivation. Przemysł Chem..

[15] Trawczyński C. (2013). Wpływ dolistnego nawożenia preparatem Herbagreen na plonowanie ziemniaków/The effect of foliar fertilization with Herbagreen on potato yielding. Ziemn. Pol..

[16] Trawczyński C. (2018). The effect of foliar preparation with silicon on the yield and quality of potato tubers in compared to selected biostimulators. Fragm. Agron..

[17] Radkowski A., Radkowska I. (2018). Effects of silicate fertilizer on seed yield in timothy-grass (*Phleum pratense* L.). Ecol. Chem. Eng. S.

[18] Artyszak A. (2018). Effect of silicon fertilization on crop yield quantity and quality—A literature review in Europe. Plants.

[19] Harland J.I., Jones C.K., Hufford C., Draycott A.P. (2006). Co-products. Sugar Beet.

[20] Savvas D., Ntatsi G. (2015). Biostimulant activity of silicon in horticulture. Sci. Hortic..

[21] Yamaji N., Sakurai G., Mitani-Ueno N., Ma J.F. (2015). Orchestration of three transporters and distinct vascular structures in node for intervascular transfer of silicon in rice. Proc. Natl. Acad. Sci. USA.

[22] Jitsuyama Y., Akihiko T., Chiharu M., Kazuto I., Ichikawa S. (2009). Endogenous components and tissue cell morphological traits of fresh potato tubers affect the flavour of steamed tubers. Am. J. Potato Res..

[23] Henriet C., Draye X., Oppitz I., Swennen R., Delvaux B. (2006). Effects, distribution and uptake of silicon in banana (*musa* spp.) under controlled conditions. Plant Soil.

[24] Crusciol C.A.C., Pulz A.L., Lemos L.B., Soratto R.P., Lima G.P.P. (2009). Effects of silicon and drought stress on tuber yield and leaf biochemical characteristics in potato. Crop Sci..

[25] Jarosz Z. (2013). The effect of silicon application and type of substrate on yield and chemical composition of leaves and fruit of cucumber. J. Elem..

[26] Castro G.S.A., Crusciol C.A.C. (2015). Effects of surface application of dolomitic limestone and calcium-magnesium silicate on soybean and maize in rotation with green manure in a tropical region. Bragantia.

[27] Zieleniewicz W., Wróbel B. (2018). Effect of differential nitrogen fertilization on the nutritive value of fodder mallow (*Malva verticillata* L.) and maize (*Zea mays* L.) Eurostar variety. J. Res. Appl. Agric. Eng..

[28] Miao B.H., Han X.G., Zhang W.H. (2010). The ameliorative effect of silicon on soybean seedlings grown in potassium deficient medium. Ann. Bot. Lond..

[29] Shahid M.A., Balal R.M., Pervez M.A., Abbas T., Aqeel M.A., Javaid M.M., Garcia-Sanchez F. (2015). Foliar spray of phyto-extracts supplemented with silicon: An efficacious strategy to alleviate the salinity-induced deleterious effects in pea (*Pisum sativum* L.). Turk. J. Bot..

[30] Guo Z.G., Liu H.X., Tian F.P., Zhang Z.H., Wang S.M. (2006). Effect of silicon on the morphology of shoots and roots of alfalfa (*Medicago sativa*). Aust. J. Exp. Agric..

[31] Liu H., Guo Z. (2011). Effects of supplementary silicon on nitrogen, phosphorus and potassium contents in the shoots of medicago sativa plants and in the soil under different soil moisture conditions. Chin. J. Appl. Environ. Biol..

[32] Yang R., Howe J.A., Golden B.R. (2018). Calcium silicate slag reduces drought stress in rice (*Oryza sativa* L.). J. Agron. Crop Sci..

[33] Makabe S., Kakuda K., Sasaki Y., Ando T., Fujii H., Adno H. (2009). Relationship between mineral composition or soil texture and available silicon in alluvial paddy soils on the Shounai plain. Jpn. J. Soil Sci. Plant Nutr..

[34] Zuccarini P. (2008). Effects of silicon on photosynthesis, water relations and nutrient uptake of *Phaseolus vulgaris* under NaCl stress. Biol. Plant..

[35] Leriche E.L., Wang-Pruski G., Zheljazkov V.D. (2009). Distribution of elements in potato (*Solanum tuberosum* L.) tubers and their relationship to after-cooking darkening. Hort. Sci..

[36] Eneji A.E., Shinobu I., Satoru M., Jing L., Taiichiro H., Ping A., Tsuji W. (2008). Growth and nutrient use in four grasses under drought stress as mediated by silicon fertilizers. J. Plant Nutr..

[37] Hernandez-Apaola L. (2014). Can silicon partially alleviate micronutrient deficiency in plants? a review. Planta.

[38] Mali M., Aery N.C. (2008). Influence of silicon on growth, relative water contents and uptake of silicon, calcium and potassium in wheat grown in nutrient solution. J. Plant Nutr..

[39] Mali M., Aery N.C. (2008). Silicon effects on nodule growth, dry-matter production, and mineral nutrition of cowpea (*Vigna unguiculata*). J. Plant Nutr. Soil Sci..

[40] IUSS Working Group WRB (2015). World Reference Base for Soil Resources 2014. International Soil Classification System for Naming Soils and Creating Legends for Soil Maps. Update 2015.

[41] PN-R-04023:1996 Agro-Chemical Analysis of Soil—Determination of Available Phosphorus Content in Mineral Soils.

[42] PN-R-04022:1996/Az1:2002 Agro-Chemical Analysis of Soil—Determination of Available Potassium Content in Mineral Soils.

[43] Artyszak A., Gozdowski D., Kucińska K. (2015). Foliar nutrition effectiveness for sugar beet cultivated as following crop after winter rape. Sugar Ind..

[44] Research Procedure of the Regional Agrochemical Station in Warsaw No. PB 59 ed.

[45] Research Procedure of the Regional Agrochemical Station in Warsaw No. PB 20 ed.

[46] Research Procedure of the Regional Agrochemical Station in Warsaw No. PB 21 ed.

[47] Research Procedure of the Regional Agrochemical Station in Warsaw No. PB 22 ed.

[48] Research Procedure of the Regional Agrochemical Station in Warsaw No. PB 23 ed.

[49] Kraska J.E., Breitenbeck G. (2010). Simple, robust method for quantifying silicon in plant tissue. Commun. Soil Sci. Plant Anal..

[50] Buchholz K., Märländer B., Puke H., Glattkowski H., Thielecke K. (1995). Neubewertung des technischen Wertes von Zuckerrben. Zuckerindustrie.

